# Ultrastructure of *Hirschmanniella diversa* early-stage infection in browning rhizomes of Indian lotus

**DOI:** 10.21307/jofnem-2020-055

**Published:** 2020-07-06

**Authors:** Shigeru Uematsu, Tetsuo Yabu, Mitsuyoshi Yao, Takayuki Kurihara, Hironori Koga

**Affiliations:** 1 Department of Bioproduction Science, Ishikawa Prefectural University, 1-308 Nonoichi, Ishikawa 921-8836, Japan; 2 Ishikawa Agriculture and Forestry Research Center, Agricultural Experiment Station, Kanazawa, Ishikawa 920-3198, Japan

**Keywords:** Digestive enzyme, *Nelumbo nucifera*, Tuber browning

## Abstract

Browning rhizome (also known as “browning tuber disease”) in Indian lotus (*Nelumbo nucifera*) caused by the nematode *Hirschmanniella diversa* is an emerging agronomic problem. In this study, the authors documented the early infection processes of *H. diversa* in the apices of young rhizomes of Indian lotus by electron microscopy analysis using an artificial inoculation method. Nematodes were attracted to young rhizome apices, invading them via narrow indentations by 4.5 hr after inoculation. Host cells adjacent to the cavity around the invading nematodes were absent and appeared to have disintegrated during infestation. Following contact with the nematodes, host cell walls and cellular contents became electron-dense and less defined, likely due to digestive enzymes secreted by the invading nematodes. Nematodes invaded to a depth of about 1 mm by 24 hr after inoculation, but did not penetrate the plant any further vertically, similar to the observation of browning in mature rhizomes in the field. The authors propose that the invasion sites of young rhizome apices become blackish-brown blotches as rhizomes mature in the field due to oxidation.

Indian lotus (*Nelumbo nucifera* Gaertn), cultivated in Japan since ancient times, includes both the flowering lotus, with high ornamental value, and the edible lotus, the rhizomes of which are consumed as a vegetable (Minamikawa and Tanaka, 1959). Mihira and Nagai (1996) reported that nematodes cause blackish-brown blotches on the surface of edible lotus rhizomes. These blotches were determined to be the symptoms of browning rhizome of Indian lotus (also known as “browning tuber of Indian lotus”), “Kurokawa-Senchu-Byo,” and the major species causing this disease was identified as *Hirschmanniella imamuri* Sher, 1968 (Mihira, 2002). Subsequently, a pathogen isolated from lotus showing the same symptoms was identified as *Hirschmanniella diversa* Sher, 1968 (Sher, 1968) by Mizukubo (2002). Currently, both *H. diversa* and *H. imamuri* are considered the major species causing browning rhizome disease in Japan, with occurrences of *H. diversa* expanding countrywide (Koyama et al., 2013; Mizukubo, 2015).

*Hirschmanniella* spp. are long and slender nematodes with a 15 to 46 μm long stylet, which is about three to five times the maximum width of the cephalic region (Sher, 1968; Siddiqi, 2000). These nematodes are well adapted to aquatic life and parasitize the roots of paddy plants and several aquatic plants. The genus presently contains 35 valid species found all over the world (Siddiqi, 2000; De Ley et al., 2007). Three species of *Hirschmanniella* have been reported in Japan: *Hirschmanniella oryzae* (van Breda de Haan, 1902) Luc and Goodey, 1964, *H. imamuri*, and *H. diversa* (Mizukubo, 2002). Both *H. oryzae* and *H. imamuri* parasitize rice (*Oryza sativa* L.) roots and cause root rot (Vecht and Bergman, 1952; Babatola and Bridge, 1980; Bilgrami et al., 1985).

Many cytological studies have investigated the attraction, parasitic behavior, and feeding behavior of *H. oryzae* and *H. imamuri* (Babatola and Bridge, 1980; Bilgrami et al., 1985; Goto, 1969; Mihira and Nagai, 1996; Vecht and Bergman, 1952), but little is known about these properties in *H. diversa* (Liu and Yu, 2002; Uematsu et al., 2015, 2016). Liu and Yu (2002) observed the morphology of *H. diversa* using scanning electron microscopy, and Uematsu et al. (2015) described three weed species parasitized by *H. diversa* in Indian lotus fields. Light and electron microscopy analysis of Indian lotus rootlets revealed all stages of the *H. diversa* lifecycle, from the egg to the juvenile and adult forms (Uematsu et al., 2016). However, the parasitic behavior of *H. diversa* and invasion of Indian lotus rhizomes, where blackish-brown blotches appear, has not been studied.

Preliminary experiments showed that *H. diversa* is strongly attracted to and penetrates the apex of young lotus rhizomes. Therefore, we aimed to clarify the early infection processes of *H. diversa* invading the apex of young lotus rhizomes and the resulting changes in host cells using light and electron microscopy. Invading nematodes move rapidly, and most move away from the surface of the apices soon after inoculation, when rhizomes are immersed in glutaraldehyde as a chemical fixative. Although rapid fixation techniques such as high-pressure freezing (Lak et al., 2015) are suitable for observing authentic host–parasite interfaces, only very limited area is used for fixation. Therefore, we froze specimens using liquid nitrogen to prevent nematode movement and then immersed the frozen specimens in chemical fixative to examine the ultrastructural interfaces.

## Materials and methods

### Indian lotus and nematode

Indian lotus “Shina-shirobana” rhizomes were obtained from fields where *H. diversa* had not been detected. Nematode inoculum was obtained from infected fresh rootlets of the same lotus cultivar harvested from fields where rhizome browning caused by nematodes has been frequently found. Rootlets were chopped into approximately 1 to 2-cm pieces. Nematodes were collected from the rootlets using the Baermann funnel method with 72 hr of incubation at 24 ± 1°C.

### Nematode inoculation

**Figure 1:**
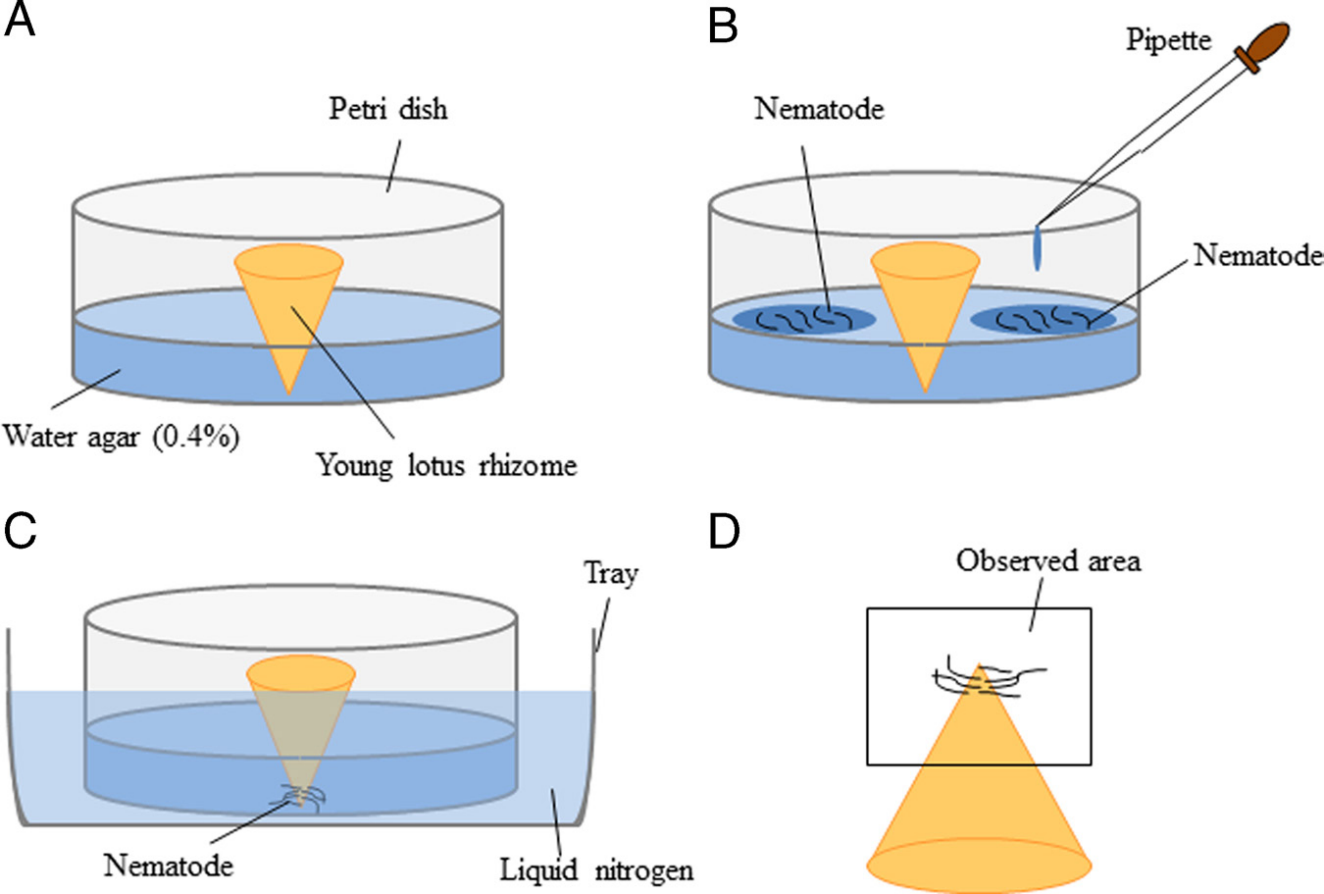
Inoculation of *H. divers*a Sher, 1968 into young lotus rhizome tissues. (A) Apices of excised young lotus rhizome tissue approximately 1 cm in length in the center of petri dishes containing 4.0 ml of 0.4% water agar. (B) Approximately 100 to 300 nematodes were added by pipette to each dish. (C) Petri dishes containing the young lotus rhizome, water agar, and nematodes were frozen in liquid nitrogen at 4.5, 24, 48, and 72 hr after inoculation. (D) Apices of young lotus rhizome tissues (*box*) were observed under a stereomicroscope after thawing and removal of water agar around the nematodes.

Apices of young lotus rhizomes (1 cm length) were excised and placed in the center of 3.0-cm-diameter petri dishes containing 4.0 ml of 0.4% water agar, with the apex in contact with the bottom of the dish (Fig. 1A, B). Approximately 100 to 300 nematodes were poured around the rhizome tissue. Petri dishes were sealed with plastic tape to prevent the water agar from drying and maintained in darkness at 24±1°C. After 4.5, 24, and 72 hr, the petri dishes of young lotus rhizomes inoculated with nematodes were frozen rapidly in liquid nitrogen (Fig. 1C). After thawing, the inoculated lotus rhizome apices were observed with stereomicroscopy. Apices were fixed for light and electron microscopy (Fig. 1D).

### Microslicer

Inoculated (or uninoculated control) specimens were immersed in 2.5% glutaraldehyde in 0.05 M cacodylate buffer, pH 7.2, for 12 to 72 hr at 4°C. Specimens were sectioned into 90-μm-thick slices with a microslicer (DTK-3000; Dosaka EM, Kyoto, Japan) and observed utilizing a light microscope.

### Scanning electron microscopy

Specimens inoculated with nematodes were immersed in 2.5% glutaraldehyde in 0.05 M cacodylate buffer, pH 7.2, for 12 to 24 hr at 4°C. Postfixation with 1% osmium tetroxide was performed in the same buffer for 12 hr at 4°C. Specimens were dehydrated in a graded series of ethanol: 50, 70, 80, 90, and 100%. Thereafter, ethanol was exchanged with 100% t-butyl alcohol (2-methyl 2-propanol), and specimens were freeze-dried (ES-2030; Hitachi, Tokyo, Japan). The specimens were coated with approximately 8 nm of platinum by ion spatter (E-1010; Hitachi) and observed by field emission SEM (S-4700; Hitachi) at 25 kV. To observe the inner tissues of rhizomes that had been invaded by nematodes, specimens were vertically cut into two pieces with a razor blade. The pieces were coated with platinum and observed by SEM as described above.

### Transmission electron microscopy

Specimens inoculated with nematodes were immersed in 2.5% glutaraldehyde in 0.05 M cacodylate buffer, pH 7.2, for 24 hr at 4°C and then postfixed in 1% osmium tetroxide in the same buffer at 4°C for 12 hr. The specimens were rinsed with distilled water for 10 min and dehydrated in an ethanol series: 50, 70, 80, 90, and 100%. Absolute 100% ethanol was then replaced with QY-1 (1-Butoxy-2, 3-epoxypropane), and specimens were embedded in Quetol 651 resin mixture (Nissin EM, Tokyo, Japan). For light microscopy, 0.5-μm-thick sections were cut using glass knives and stained with 1% toluidine blue in 1% sodium borate solution. For transmission electron microscopy, ultrathin sections were cut from resin blocks with an ultramicrotome (EM UC6; Leica, Vienna, Austria) using a diamond knife. Ultrathin sections were collected on 200 × 75mesh Formvar-coated grids, stained with saturated uranyl acetate in distilled water for 10 min, and then stained with lead citrate for 10 min. A Hitachi H-7650 transmission electron microscope was used to observe the interfaces between invading nematodes and host cells.

## Results

### Stereomicroscopy

**Figure 2:**
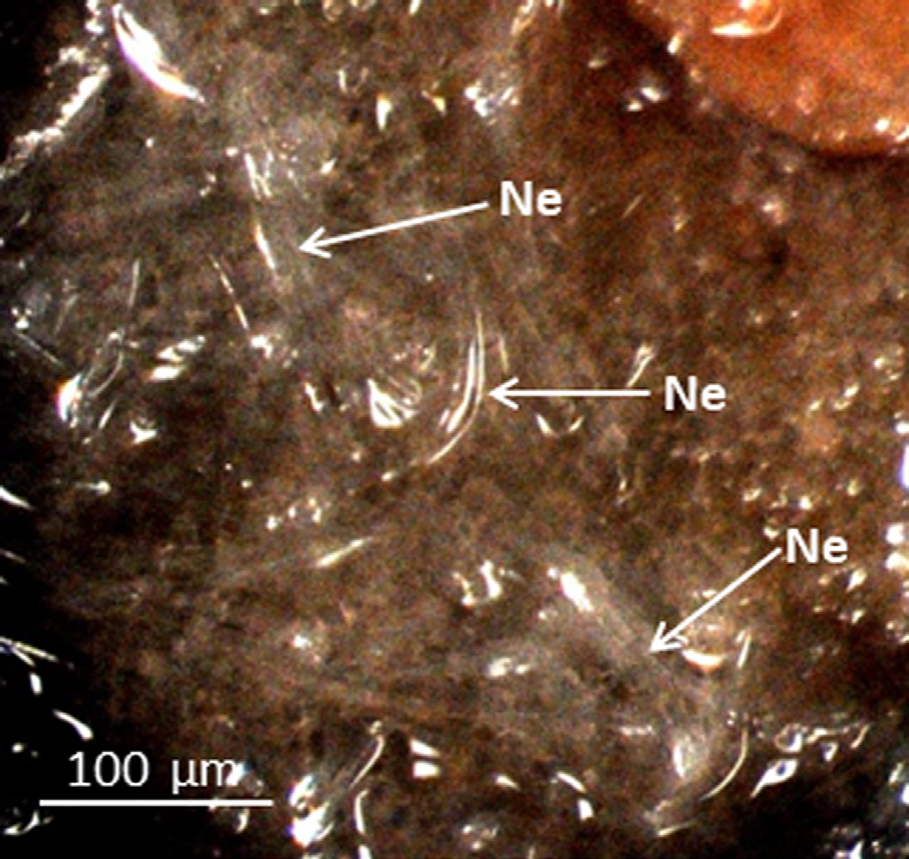
Stereoscope micrograph of *H. diversa* Sher, 1968 invading the apex of a young lotus rhizome 4.5 hr after inoculation. Note that many nematodes remained at the apex of the rhizome even after the water agar was removed. Ne, nematode.

Nematodes began to move towards the apices of young rhizomes when nematode suspensions were poured into the surrounding water agar in petri dishes. After 4.5 hr of inoculation, many nematodes gathered around the surface of the apices and invaded them (Fig. 2). After thawing the agar, most of the nematodes remained attached to the apices when the agar around the nematodes was removed. At both 24 and 72 hr after inoculation, the number of nematodes invading the apices was much higher than at 4.5 hr after inoculation.

### Light microscopy

**Figure 3:**
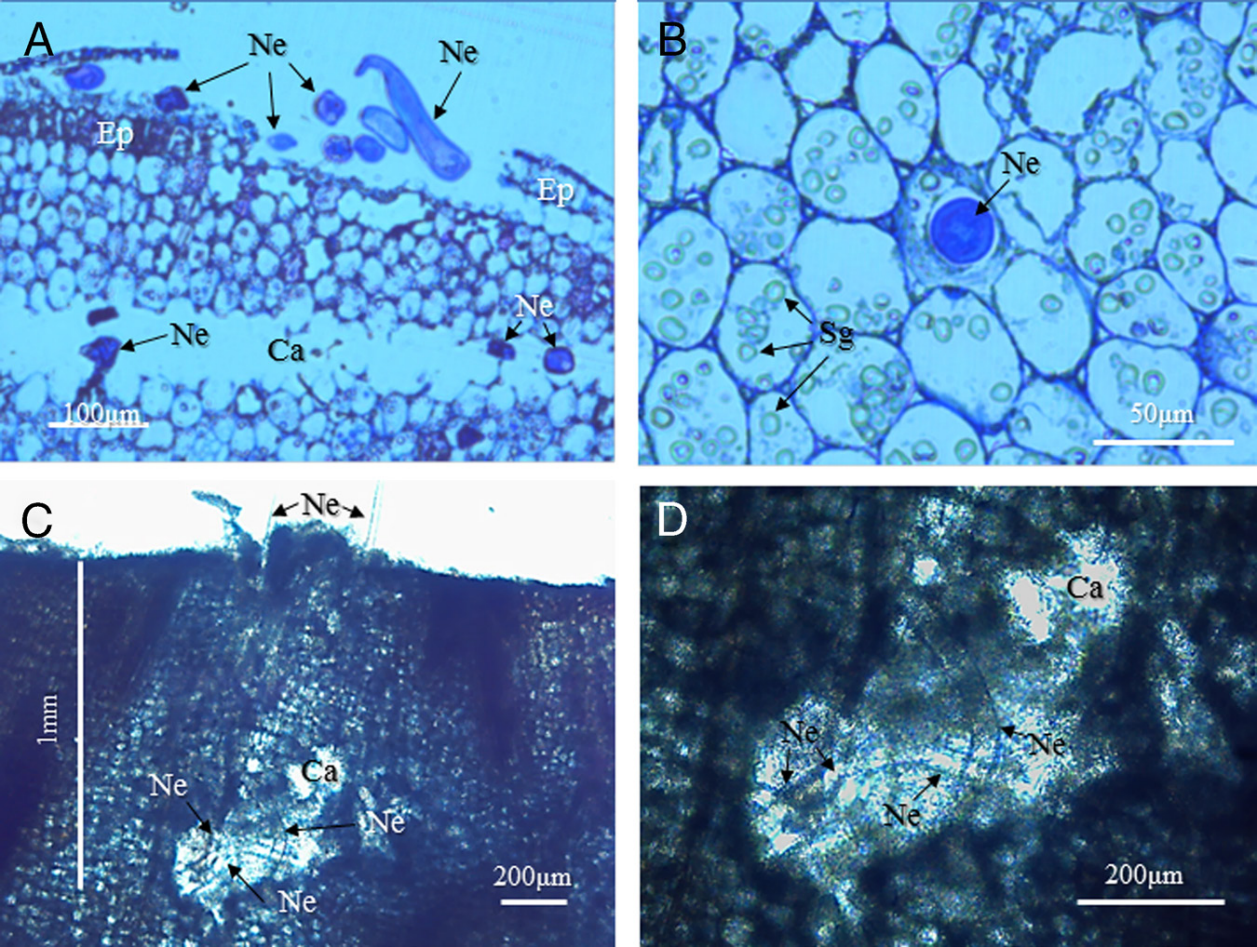
Light micrographs showing cross sections of the apices of young rhizomes inoculated with *H. diversa* Sher, 1968 at 24 hr (A, B) and 72 hr (C, D) after inoculation. (A) Young rhizome apex invaded by nematodes. Note that nematodes (Ne) invaded the epidermis (Ep) and the cavity (Ca), and several layers of cortical cells disappeared. (B) Cortical cell invaded by a nematode. Note that the host cell wall collapsed in the cell invaded by the nematode. (C) Young rhizome invaded by nematodes. Note that invading nematodes stopped penetrating in a vertical direction relative to the surface at about 1 mm depth from the surface. (D) Magnification of Fig. 3C, box. Host tissues surrounding the invading nematodes disappeared and cavities were observed.

We observed many invading nematodes in the epidermis and in a cavity where a few layers of cortical cells had disappeared at 24 hr after inoculation (Fig. 3A). Nematode invasion of some cortical cells was also observed. The host cell wall appeared to have collapsed in the cell invaded by the nematode (Fig. 3B). In total, 72 h after inoculation, nematodes had penetrated to a depth of about 1 mm from the surface (Fig. 3C). The host tissues surrounding the invading nematodes had disappeared and cavities were observed (Fig. 3D).

### Scanning electron microscopy

**Figure 4:**
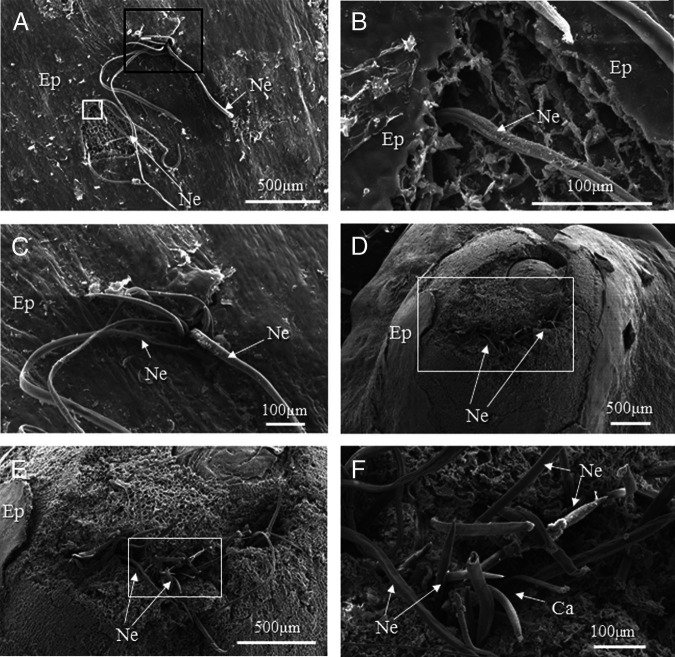
SEM secondary electron images of the surfaces of young lotus rhizome apices invaded by *H. diversa* Sher, 1968 at 4.5 hr (A-C) and 24 hr (D-F) after inoculation. (A) Nematodes (Ne) invading the epidermis (Ep) of the apex. (B) Magnified micrograph of the white box in Fig. 4A. The forepart of the nematode burrowed under the peeled part of the epidermis of the apex. (C) Magnified micrograph of the black box in Fig. 4A. Clusters of nematodes penetrated the epidermis of the apex through narrow indentations. (D) Clusters of nematodes on the tissues where the epidermis was peeled off accompanied by larger indentations than those seen at 4.5 hr after inoculation. (E) Magnified micrograph of the white box in Fig. 4D. A few clusters of nematodes have invaded the tissues from the peeled part of the epidermis. Note that host tissues around invading nematodes disappeared into indentations. (F) Magnified micrograph of the box in Fig. 4E. Only the posterior parts of most nematodes were observed, suggesting that they had penetrated the tissues deeply.

Nematodes penetrating the epidermis were observed 4.5 hr after inoculation (Fig. 4A). We observed two patterns of penetration into the apex of the young rhizome: burrowing under the peeled part of the epidermis (Fig. 4B) and direct penetration of the epidermis through narrow indentations (Fig. 4C). In the latter case, several nematodes penetrated the epidermis from the same site. In total, 24 hr after inoculation, we observed several clusters of nematodes on the tissues where the epidermis was peeled off, accompanied by large indentations around the nematodes (Fig. 4D). The indentations were larger than those at 4.5 hr after inoculation, and neighboring indentations appeared to be connected (Fig. 4E). Most nematodes had invaded more deeply into the tissues than at 4.5 hr after inoculation (Fig. 4F).

**Figure 5:**
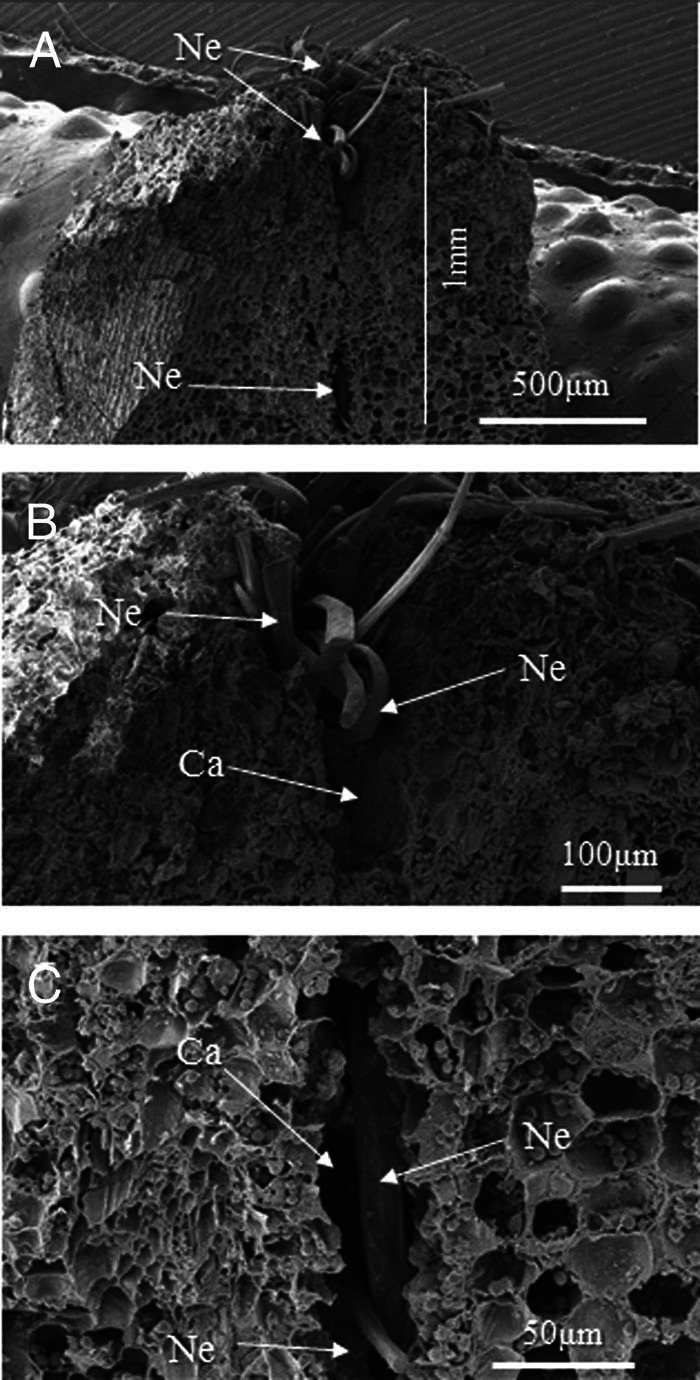
SEM secondary electron images of cross sections of the site shown in Fig. 4E, where a cluster of nematodes invaded the inner apex of a young rhizome. (A) Cross section of the apex of a young rhizome invaded by nematodes (Ne). Note that the nematodes invaded the tips of the tissue to depths of up to about 1 mm. (B) Magnified micrograph of the upper region of the young rhizome shown in Fig. 4A. A cluster of nematodes invaded the apex tissue of a young rhizome. Note that a cavity (Ca) formed around the invading nematodes. (C) Magnified micrograph of the lower region of the young rhizome shown in Fig. 4A. Host tissues at the sides of the cavity around the invading nematodes disappeared as if they had disintegrated.

SEM images of cross sections of nematodes reached a depth of at most 1 mm by 24 hr after inoculation (Fig. 5). We observed cavities in the host tissues of up to 180 μm in diameter around the invading nematodes (Fig. 5B). Host cells at the sides of the cavity had disappeared, as if they had disintegrated (Fig. 5C).

### Transmission electron microscopy

**Figure 6:**
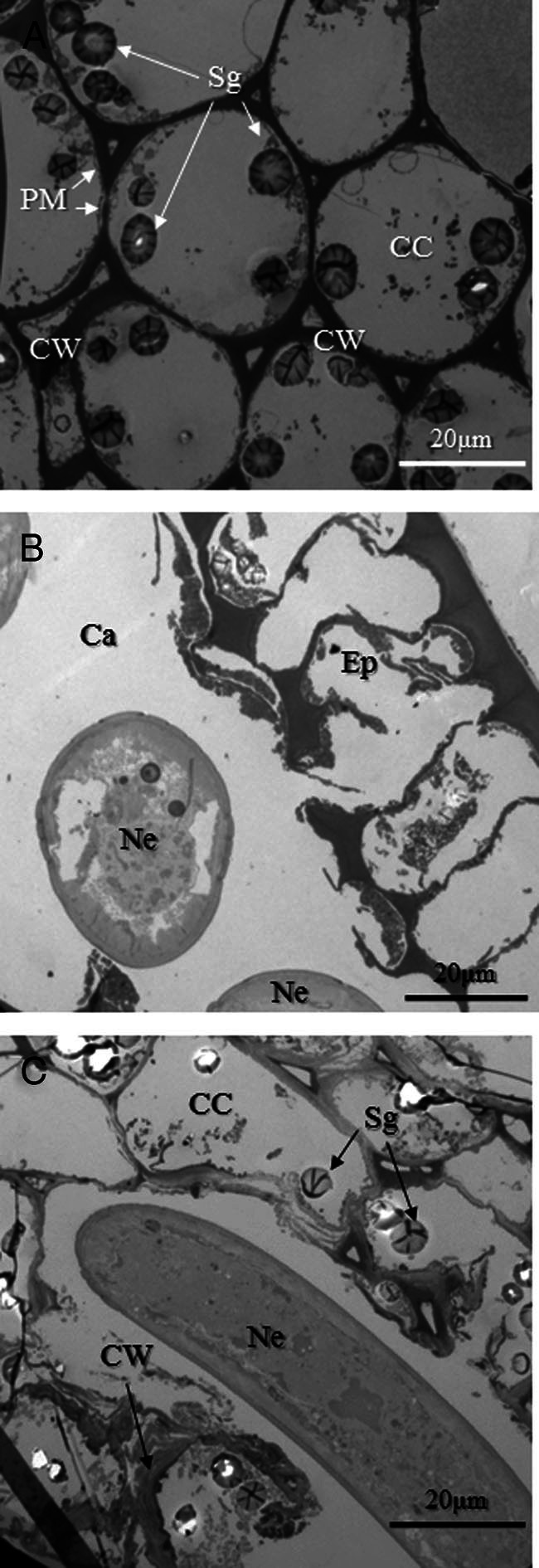
Cross section through the apex of a young rhizome inoculated with *H. diversa* Sher, 1968 at 24 hr after inoculation. (A) uninoculated cortex (control). Note that artifacts generated by freezing specimens in liquid nitrogen were observed as fragmentation of plasma membranes and collapse of cytoplasm, while cell walls (CW) and starch grains (Sg) of cortical cells (CC) show minimal damage. (B) Cross section through the epidermis (Ep) of an apex and invading nematodes (Ne). Epidermal cells neighboring the invading nematodes degraded and collapsed, and the area surrounding the nematodes formed a cavity (Ca). (C) Cross section of a cortex invaded by a nematode. The cortical cells close to the nematode disappeared, and the surrounding host cells degraded.

Artifacts generated by freezing in liquid nitrogen during sampling included the collapse of the cytoplasm and fragmentation of plasma membranes in uninoculated apices (Fig. 6A). However, artifacts were minimal on the cell walls and starch grains of host cells (Fig. 6A). By contrast, the epidermis and cortex around invading nematodes had degraded and were electron-dense and partly dissolved, suggesting that the nematodes had digested host tissues enzymatically (Fig. 6B, C). The area surrounding invading nematodes was clear (Fig. 6B, C).

**Figure 7:**
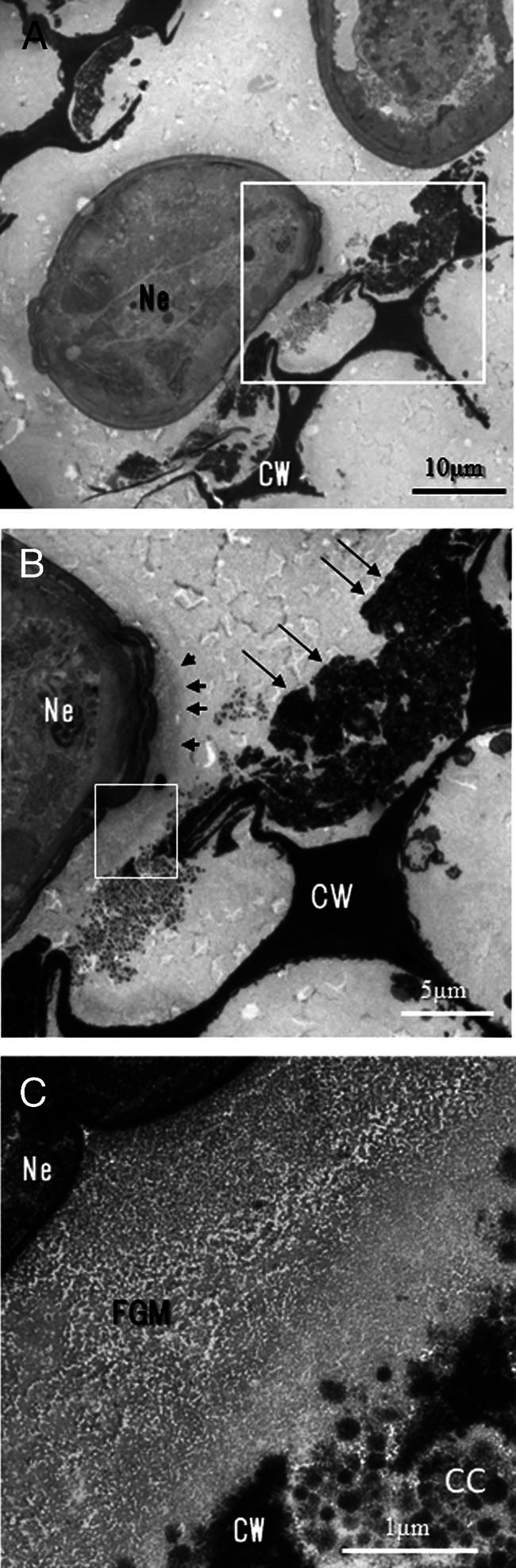
Cross section through the cortex of a young rhizome apex invaded by nematodes. (A) Cortical cells close to invading nematodes (Ne). Note that the upper portions of cortical cells disappeared, and the cell wall (CW) and cytoplasm of the remaining cell degraded and became electron-dense. (B) Magnified micrograph of the box in Fig. 7A. Weakly electron-dense materials (thick arrowheads) are observed close to the nematode. Note that the host cells close to the nematode disappeared linearly (thin arrows). (C) Magnified micrograph of the box in Fig. 7B. The weakly electron-dense materials are composed of fine granular materials (FGM), which are denser closer to the nematode. Note that outlines of the cell wall (CW) and the cellular content (CC) are indistinct.

Cell walls facing the cavities were degraded and sometimes completely absent (Fig. 7A). Remaining cell walls and cell contents of the remaining portions were electron-dense (Fig. 7B). We observed fine granular materials around the invading nematode, which appeared to be denser closer to the nematode, suggesting that they were caused by secretions from the nematode (Fig. 7B, C). The outlines of the cell walls and cellular contents became indistinct at sites adjacent to the fine granular materials (Fig. 7C).

## Discussion

We documented the early infection processes of *H. diversa* in the apices of young rhizomes of Indian lotus by use of an artificial inoculation method. Most of the nematodes were attracted to the apices of young rhizomes within a short period and invaded the rhizomes. Vecht and Bergman (1952) reported that multiple *Radopholus oryzae* (*H. oryzae*) nematodes invaded roots of rice plants through previous nematode entry points. Such an invasion mode was also observed in the root-knot nematode with light microscopy and SEM (Wergin and Orion, 1981; Sijmons et al., 1991). We suggest that nematode-attractive compounds are leaking from epidermis already damaged.

Uematsu et al. (2016) suggested that digestive enzymes secreted by *H. diversa* caused the formation of cavities in the cortex of Indian lotus rootlets. Based on our TEM analysis of migration through the young rhizome, we hypothesize that digestive enzymes secreted by nematodes degrade host tissues, causing cell walls and cellular contents to become electron-dense, and finally digested altogether, to create indentations at the nematode penetration sites and cavities around the invading nematodes. Our SEM analysis further indicated that host tissues around the invading nematodes had disappeared linearly. Babatola and Bridge (1980) reported detailed comparisons of the feeding behavior of *H. oryzae*, *H. imamuri*, and *H. spinicaudata* on rice. In these three species, the ampula of the dorsal oesophageal gland duct enlarged during salivation, followed by a continuous flow of secretions through the stylet into the punctured cell. Zunke (1990) reported the endoparasitic behavior of the root-lesion nematode, *Pratylenchus penetrans*, using video-enhanced contrast microscopy to observe living nematodes. After penetration, a small (about 2 μm thick) “salivation zone” was formed around the stylet tip, and granules from the dorsal esophageal gland flowed down the gland duct to the ampulla, which opens just behind the stylet knob during salivation. In our study, we observed with TEM a layer of fine granular materials, about 2 μm in thickness, close to the nematode. We propose that this region is the salivation zone reported for *P. penetrans* at the cell penetration site (Zunke, 1990), although it remains to be confirmed that this zone formed around the stylet tip.

Since *H. diversa* is a migratory endoparasite, such destructive digestion may be effective not only for obtaining nutrients within a short period of time, but also for avoiding host responses. This infection process differs from the dissolution of cell walls observed in both sedentary endoparasitic cyst nematodes (Sobczak and Golinowski, 2009) and root-knot nematodes (Paulson and Webster, 1970; Hussey and Mims, 1991; Berg et al., 2009; Miyashita and Koga, 2017) after they begin to form syncytia and giant cells, respectively.

If root pieces are not frozen in liquid nitrogen prior to 4.5 hr after inoculation, most of the penetrating nematodes separate from the surfaces of host tissues upon immersion in chemical fixative. In addition, fine structures of host tissues penetrated by nematodes might be partly destroyed due to the movement of invading nematodes in response to the chemical. Hussey and Mims (1991) observed that the slow action of chemical fixatives precluded electron microscopy observations of interfaces between nematode stylets and the lumen of the feeding tube in giant cells induced by root-knot nematodes. Liquid nitrogen treatment before fixation with chemical fixatives caused fragmentation of plasma membranes and destruction of the cytoplasm due to physical force. By contrast, damage to host tissues caused by invading nematodes was mainly from chemical reactions such as lysis and the degeneration of the host cell wall and cytoplasm, accompanied by an increase in electron density. Therefore, the damage caused by liquid nitrogen treatment could be distinguished from that caused by nematode invasion. Artifacts caused by liquid nitrogen treatment were minimal in host tissues that had already been degraded by nematode invasion.

Uematsu et al. (2015) reported that *H. diversa* parasitized roots of seven species belonging to four plant families. Therefore, we assume that *H. diversa* has a strong tendency to parasitize a wide range of plant roots, even young rhizomes. Vecht and Bergman (1952) and Goto (1969) reported that the rice nematode *Radopholus oryzae* (*= Hirschmanniella imamuri*, *H. oryzae*) invades the roots of rice plants and lives in the cavities between the parenchyma lamellae of the cortex. *Hirschmanniella diversa* lives in root cortex cells for almost all their lives; in contrast, when invading the apices of young rhizomes, the nematode does not penetrate beyond a depth of about 1 mm. We presume that this difference is due to different conditions; the cell walls of roots were markedly thinner and softer than those of young rhizomes, and the volumes of cortical cells were more than eight times larger (Uematsu et al., 2016).

Blackish-brown blotches characteristic of rhizome browning in mature rhizomes were at most 1 mm deep, and no nematodes were observed there (Uematsu, unpublished data), corresponding to the depth at which invading nematodes stopped progressing in the apices of young rhizomes in this study. In addition, nematodes can burrow between the epidermis and cortex in mature rhizomes, but cannot invade cortical cells (Uematsu, unpublished data). Therefore, the blackish-brown blotches observed in mature rhizomes in the field appear to arise from the invasion sites at the young rhizome apices and change color due to oxidation (Nagashima et al., 1997). We suggest that the cortical cells in the apices of young rhizomes are so unfavorable that nematodes escape from the invasion sites. Further studies to verify that these invasion sites indeed develop into the symptoms of rhizome browning in the field are currently in progress.
